# The Prognostic and therapeutic value and clinical implications of fibroblast activation protein-α as a novel biomarker in colorectal cancer

**DOI:** 10.1186/s12964-023-01151-y

**Published:** 2023-06-14

**Authors:** Zahra Kalaei, Reyhaneh Manafi-Farid, Bentolhoda Rashidi, Fariba Karoon Kiani, Asieh Zarei, Mehrdad Fathi, Farhad Jadidi-Niaragh

**Affiliations:** 1 Department of Biology, Faculty of Natural Sciences, Tabriz University, Tabriz, Iran; 2 Research Center for Nuclear Medicine, Tehran University of Medical Sciences, Tehran, Iran; 3 Immunology Research Center, Tabriz University of Medical Sciences, Tabriz, Iran; 4 Research Center for Integrative Medicine in Aging, Aging Research Institute, Tabriz University of Medical Sciences, Tabriz, Iran; 5 Department of Immunology, Faculty of Medicine, Tabriz University of Medical Sciences, Tabriz, Iran

**Keywords:** Fibroblast activation protein, Cancer-associated fibroblasts, Tumor microenvironment, Colorectal cancer

## Abstract

The identification of contributing factors leading to the development of Colorectal Cancer (CRC), as the third fatal malignancy, is crucial. Today, the tumor microenvironment has been shown to play a key role in CRC progression. Fibroblast-Activation Protein-α (FAP) is a type II transmembrane cell surface proteinase expressed on the surface of cancer-associated fibroblasts in tumor stroma. As an enzyme, FAP has di- and endoprolylpeptidase, endoprotease, and gelatinase/collagenase activities in the Tumor Microenvironment (TME). According to recent reports, FAP overexpression in CRC contributes to adverse clinical outcomes such as increased lymph node metastasis, tumor recurrence, and angiogenesis, as well as decreased overall survival. In this review, studies about the expression level of FAP and its associations with CRC patients' prognosis are reviewed. High expression levels of FAP and its association with clinicopathological factors have made as a potential target. In many studies, FAP has been evaluated as a therapeutic target and diagnostic factor into which the current review tries to provide a comprehensive insight.

Video Abstract

**Supplementary Information:**

The online version contains supplementary material available at 10.1186/s12964-023-01151-y.

## Introduction

Annually, 900,000 deaths from Colorectal Cancer (CRC) are reported, and 1.8 million new patients are diagnosed with this cancer. The incidence of CRC is increasing gradually in the world and it is predicted that by 2035, the number of new cases will reach 2.5 million worldwide [1, 2]. CRC is the third leading cause of cancer death worldwide [3]. The risk factors for CRC are inappropriate diet, obesity, tobacco consumption [4], Gastrointestinal (GI) tract chronic inflammation, and genetic predisposition [5]. The survival of CRC patients has recently been elevated due to the screening methods in developed countries; however, 25% of patients represent stage IV at the diagnosis. Additionally, 25–50% of patients will suffer from metastatic CRC (mCRC) over time [6]. Compared to localized CRC, the 5-year survival rate of mCRC is low at 14–20% [7, 8].

Radical surgery or surgery followed by postoperative chemotherapy and radiotherapy are the primary therapeutic strategies for patients diagnosed at stages II and III [9, 10]. However, a hypothesis suggests that surgery on a primary tumor provides a good breeding ground for tumor cells and can cause tumor recurrence or increase the rate of liver metastasis [11]. In mCRC patients, surgery is the primary option; however, approximately 20–30% of cases undergo surgery [8]. Three main regimens exist in the CRC chemotherapy setting: 1) 5-fluorouracil (5-FU) and oxaliplatin, 2) 5-FU and leucovorin and irinotecan, and 3) oxaliplatin and capecitabine [12]. When a patient receives chemotherapy drugs, complications such as gastrointestinal mucositis and drug resistance may occur due to chemotherapy or nephrotoxicity [11]. These complications are barriers to achieving maximum clinical benefits in patients [13]. In addition, peripheral neuropathy and intestinal dysfunction may occur due to prolonged chemotherapy and radiotherapy and may cause other complications such as frequency and urgency [14]. Considering the exclusive complication of each CRC treatment, we need specific progress to solve the complication and develop more effective treatment approaches [6]. Moreover, it is necessary to identify the biomarkers involved in CRC growth and increased rate of metastasis and recurrence that affect prognosis and consider them novel molecular therapeutic targets discovering developing new drugs for CRC.

It has been demonstrated that Tumor Microenvironment (TME) or tumor stroma around solid tumors could be a proper platform for tumorigenesis and progression [15]. The TME consists of different kinds of cells and extracellular factors, which can play a functional role in pathological conditions besides physiological conditions [16]. Metabolic reprogramming within the TME has been proven intimately involved in the initiation and malignant progression of CRC. Signal messengers, including cytokines, metabolites, and exosomes, derived from cancer cells can be utilized by the surrounding cells within the TME to induce metabolic alteration and cancer-associated transformation. In turn, the cargos secreted from cancer-associate cells further provide the nutrition and energy supply for cancer cells, supporting their metabolic reprogramming to promote proliferation, migration, metastasis, and radiochemoresistance [17]. The TME has a dynamic composition including various cell types, such as cancer-associated fibroblasts (CAFs), tumor-associated macrophages, regulatory T cells, and myeloid-derived suppressor cells, as well as extracellular factors surrounding cancer cells which has functional and structural roles under physiological and pathological conditions [18].

One of these cell types is CAFs [19]. Interestingly, Hypoxia creates the conditions necessary for cancer growth by transforming fibroblasts into cancer-associated fibroblasts (CAFs) by disrupting the extracellular matrix and angiogenesis processes, which aid in tumor metastasis [20]. The term “CAF” is generally used to describe the activated (i.e., no longer quiescent) fibroblastic cell population that accompanies solid epithelial tumors [21]. CAFs are stromal fibroblastic cells that undergo phenotypic and functional changes and regulate many tumorigenic processes [22–24]. CAFs are recognized as microenvironmental cells that provide metabolic support to cancer cells [25, 26]. CAFs are also immuno-modulatory cells with immunosuppressive and immunogenic functions [22].

According to the investigations, CAFs participate in tumorigenesis in different ways. They promote angiogenesis, increase proliferation and invasion, and suppress the immune system [27]. There are a lot of CAFs in the tumor microenvironment, and these CAFs have a significant impact on how CRC progresses. Theoretically, focusing on CAFs has a lot of potentials to improve CRC treatment [28]. CAFs presence has been confirmed in other cancers such as breast, liver, and prostate [15]. CAFs can facilitate tumor cell invasion by producing Extracellular Matrix (ECM) components, particularly fibrillar collagens, as well as a wide range of CAFs-released growth factors and cytokines in TME. As a result, CAF can alter tumor stroma and trigger a desmoplastic reaction in TME. These alternations are associated with poor prognosis in many carcinomas [29]. In CRC, an abundance of CAFs in the TME has been associated with poor outcomes, and transcriptomic studies linked CAF signature with poor prognosis and highly aggressive CRC molecular subtypes. Transcriptome and proteome profiling identified CRC CAFs as the main source for connective tissue components of the ECM, such as collagens, which alter the matrix's molecular composition by increasing the deposition of new matrix components [30]. CAFs have several markers, including alpha-smooth muscle actin [31], tenascin-C, platelet-derived growth factor receptor-alpha/beta, CD90 [32], podoplanin [33], vimentin, desmin, fibroblast-specific protein 1 [34], and Fibroblast-Activation Protein (FAP) [35].

FAP plays an important role in tissue remodeling and aids tumor cells development by multiple mechanisms, including immunosuppression, drug resistance, stem cell promotion, promoting invading surrounding tissue, proliferation, angiogenesis, and epithelial-to-mesenchymal transition [36]. As a result, cancer patients with high levels of FAP expression have worse clinical outcomes. Most of FAP's functions have been linked to its enzymatic activity in tissue remodeling, which aids tumor cells in invading surrounding tissue, breaking through the wall of blood vessels, and traveling to form distant metastases [37–40]. FAP was found in more than 93% of CRC tumors, according to earlier investigations. Thirty percent of those displayed intense FAP staining [41]. In metastatic CRCs, high FAP expression has been suggested as a biomarker for disease progression [42, 43]. FAP has been suggested as a potential option for targeted therapy in CRC based on the available scientific data. FAP is a type II transmembrane cell surface proteinase [4] and belongs to Dipeptidyl Peptidase (DPP) family. It consists of four enzymes DPP4, FAP, DPP8, and DPP9 [44]. FAP has 760 amino acids and bounds to the plasma membrane via the twenty amino acids forming a signal sequence of FAP. FAP has an amino-terminal and cytoplasmic domain constructed by six amino acids [45]. In monomer form, FAP is inactive and must undergo dimerization to conduct enzymatic activity [15]. In a homodimeric 170 kDa form, FAP enzymatic functions include di- and endo-prolylpeptidase activities [40]. As a polypeptide, FAP removes dipeptides from the N-terminal domain of polypeptides, which contains proline or alanine in their penultimate position [46]. Like prolyl oligopeptidase, FAP also possesses endoprotease activity [36] and gelatinase/collagenase functions [47]. FAP’s endopeptidase activity is a unique function that distinguishes it from other members of the DPP4 family. Hamson et al*.* stated that this seems to be the main enzymatic role of FAP. Some investigations identified FAP’s endopeptidase activity substrates, including denatured type I collagen, α2-antiplasmin [48], gelatin, neuropeptides (e.g., neuropeptide Y), and B-type natriuretic peptide. Based on evidence about the association of FAP expression and microvessel density in tumors, Lindner et al. suggested that FAP could also participate in tumor angiogenesis [49]. FAP also possesses non-enzymatic activities. By conducting quantitative immunoprecipitation combined with knockdown (QUICK) analysis, it was demonstrated that FAP takes part in the lipid raft of the membrane and has a specified role in stromal invadopodia that eventually leads to matrix remodeling [50].

Nowadays, investigations have demonstrated the effects of FAP overexpression on tumorigenesis and the disease prognosis. Now it has been confirmed that the overexpression of FAP in several cancers such as breast [45, 51], gastric [52–54], melanoma [54, 55], and fibrosarcoma [56], can increase cell migration, invasion, differentiation, and growth, as well as angiogenesis [57]. Also, FAP overexpression affects survival by suppressing lymphocyte-dependent immune reactions in non-small cell lung cancer and pancreatic adenocarcinoma [58–60]. Regarding CRC, in metastatic patients with high FAP expression, disease progression is faster compared to those with low FAP expression [41]. Furthermore, the crucial prognostic role of FAP overexpression in CRC and its effects on survival and clinicopathological factors have been confirmed [38, 61–64]. The present study will review the investigations depicting the expression level of FAP in CRC tumoral tissue compared to normal mucosa and the potential role of FAP as a theranostic agent in CRC treatment and diagnosis.

### FAP functions in tumorigenesis

High expression of FAP is regulated via different transcription factors such as early growth response (EGR-1) and occurs in wound healing, inflammation such as arthritis, artherosclerotic plaques, and fibrosis as well as in ischemic heart tissue after myocardial Infarction and in more than 90% of epithelial carcinomas [49]. Some investigations revealed that FAP overexpression increases tumor angiogenesis in breast and gastric cancer [45, 51–54, 57]. In glioblastoma (one of the fatal cancers of the central nervous system) [65], it is revealed that FAP-positive mesenchymal cells express pro-angiogenic factors. However, compared to normal pericytes, they exhibit decreased levels of antiangiogenic molecules and an increased Angiopoietin 2/1 ratio. FAP-positive mesenchymal cells promote angiogenesis, glioma cell migration, and growth by paracrine communication, which leads to glioblastoma progression [66].

Similarly, FAP promotes CRC angiogenesis via the Akt and ERK signaling pathways [67]. Also, FAP implies pro-angiogenic properties in osteosarcoma by promoting vascular endothelial growth factor-A (VEGF-A) expression. It is suggested that FAP regulated VEGF-A expression in osteosarcoma cells via the PI3K/AKT and ERK signaling pathways [68]. FAP-positive CAFs are the major source of C–C motif chemokine ligand 2 (CCL2) which can promote tumor growth by enhancing the recruitment of myeloid-derived suppressor cells. Additionally, FAP regulates tumor growth and invasiveness by increasing angiogenesis and reducing the immune system's antitumor response mediated by the STAT3/CCL2 signaling pathway [69].

FAP overexpression also promotes tumor growth in some cancers [52–54, 70]. FAP overexpression also resulted in more disease progression compared to low FAP expression in CRC [41, 71]. In glioblastoma, FAP has been proven to promote tumor growth and invasion via hydrolysis of molecules such as brevican in the extracellular matrix and targeting downstream pathways and substrates, such as fibroblast growth factor 21 (FGF21) [72]. In gastric cancer, stromal FAP promotes cancer progression via epithelial-mesenchymal transition (EMT) through Wnt/β-catenin signal pathway [60]. In oral squamous cell carcinoma, an in vitro investigation showed that FAP overexpression increases cancer cell proliferation, migration, and invasion through PTEN/PI3K/AKT and Ras-ERK activation and its downstream signaling [73].

It has been revealed that overexpression of FAP is associated with increased tumor cell migration and invasion in gastric cancer, melanoma, and osteosarcoma [52, 55, 74]. A clinical and in vivo study showed that high expression of FAP in osteosarcoma is significantly associated with the tumor size and clinical stage. They revealed that the knockdown of FAP remarkably blocked the proliferation, migration, and invasion of osteosarcoma cells in vitro*,* suppressing mouse tumor growth and metastasis via the AKT signaling pathway [75]. FAP promotes the growth, adhesion, and migration of lung Squamous Cell Carcinoma. Also, FAP regulates lung cancer cell function, potentially via the PI3K and SHH pathways [76]. Kawase et al*.* examined the effect of FAP-expressing fibroblasts on invasiveness and the cell cycle in MiaPaCa-2 cells (a pancreatic cancer cell line). They found that FAP-expressing fibroblasts promoted the invasiveness of MiaPaCa-2 cells more intensively than fibroblasts not expressing FAP. Co-culture with FAP-expressing fibroblasts significantly activated cell cycle shift in MiaPaCa-2 cells compared to fibroblasts without FAP expression. Furthermore, co-culture with FAP-expressing fibroblasts inactivated retinoblastoma (Rb) protein, an inhibitor of cell cycle progression in MiaPaCa-2 cells by promoting phosphorylation of Rb [77].

In non-small cell lung cancer and pancreatic adenocarcinoma, FAP overexpression suppressed lymphocyte-dependent immune reactions and reduced survival rate [58–60]. In a clinical investigation, stromal FAP expression was evident in most Esophageal Squamous Cell Carcinoma (ESCC) samples but was absent in adjacent normal tissue. The proportion of samples positive for stromal FAP expression was significantly higher in lymph node metastasis (N1–3) as compared to primary tumors (N0), suggesting that FAP-expressing stroma might be essential during ESCC progression [78]. Moreover, it has been reported that high FAP expression in pancreatic cancer patients resulted in lymph node metastasis and shorter survival. Pancreatic cancer cells released Transforming growth factor beta 1 (TGFβ1) and induced Pancreatic stellate cells (PSCs) to express FAP. FAP + PSCs released the chemokine (C-X-C motif) ligand 1 (CXCL1) and promoted the phosphorylation of the tyrosine kinase receptors EphB1 and EphB3 in pancreatic cancer cells. CXCL1, EphrinB1, and EphrinB3 worked together to promote the migration and invasion of pancreatic cancer cells by Akt phosphorylation [79]. Recently, an indirect co-culture model and a mixed xenograft of breast cancer demonstrated that TGF-β1-activated CAFs promote tumor invasion, pulmonary metastasis, and EMT, which act through autophagy and overexpression of FAP in both models.

In contrast, autophagy inhibitor 3-methyladenine suppresses these effects. In addition, the knockdown of FAP resulted in reversed EMT and abolished tumor invasion and pulmonary metastasis induced by TGF-β1-activated CAFs. In other words, autophagy and FAP are required for breast cancer cell invasion and metastasis [80]. Generally, FAP can cause tumor progression and metastasis; however, this review will specifically discuss FAP functions in CRC.

### Expression of FAP in cancer cells and its targeting approaches

In human lung adenocarcinoma, it has been shown that stromal FAP overexpression is associated with the worst patient prognosis [81]. In high-grade serous ovarian cancer, it was found that FAP overexpression is correlated with poor Overall Survival (OS), Progression-Free Survival (PFS), and more advanced disease stages. Based on the reports, FAP affects HGSOC prognosis through the FN1 pathway [82]. In clear renal cell carcinoma, FAP overexpression correlates with tumor aggressiveness and poor survival [83]. Also, FAP overexpressed in tumoral tissue compared to normal brain tissue, and its overexpression was associated with disease progression in glioblastoma. These findings suggest that FAP could be a novel immunological target for targeting tumor cells and the vascular network that supplies these cells [84].

It has been revealed that FAP is overexpressed in Gastrointestinal (GI) tumors. Kawase et al*.* conducted immunohistochemistry (IHC) in 48 pancreatic ductal adenocarcinoma tumor samples and found that FAP is expressed in 98% of specimens. They concluded that FAP overexpression is associated with lower cumulative survival rates [77]. It was found that FAP is overexpressed in pancreatic cancer stromal tissues compared to normal tissues by using tissue microarray analysis. In addition, pancreatic stellate cells mainly undergo FAP overexpression by inducing pancreatic cancer cells-released TGFβ1.

Moreover, FAP-positive pancreatic stellate cells are associated with more lymph node metastasis and poorer survival [79]. Gao et al*.* evaluated FAP expression in 110 Gastric Cancer (GC) samples and found 61.8% of specimens as FAP positive. In their conclusion, FAP overexpression was associated with the development of angiogenesis and increased metastasis rate [62]. FAP expression was also assessed in 112 GC tumor specimens, and it was revealed that 62.5% of patients had high FAP expression associated with primary tumor invasion and high TNM stage. Moreover, high FAP expression correlated with poorer overall and progression-free survival [85]. Another IHC assay investigation on 105 GC tissue samples reported that FAP overexpression in GC patients was associated with poor patient survival [86].

Ha et al*.* conducted IHC assays on 116 ESCC samples to evaluate CAF markers expression in ESCC. They found that immature CAFs showed higher expression of CAF markers, including FAP, and immature CAFs in the tumor stroma promoted epithelial to mesenchymal transition. Based on their findings in 61.2% of samples, FAP was highly expressed and was associated with a shorter 5-year overall survival rate [63]. Another study on 94 ESCC tissue samples found that FAP was expressed in approximately 53% of samples. Compared to the low FAP expression group, its overexpression significantly correlated with higher lymph node metastasis [61]. FAP expression was also evaluated in 138 Hepatocellular Carcinoma (HCC) samples via IHC. Approximately 46.3% of cases had high FAP expression, which was correlated with poorer overall survival compared to low FAP expression. Furthermore, in hypoxia conditions, FAP overexpression caused poor prognosis in these patients [87].

Considering the above studies, FAP can be considered a novel therapeutic target due to its exclusive enzymatic activity and selective expression in the tumor stroma [88, 89]. It could be targeted in different ways, one of which is utilizing Chimeric Antigen Receptor (CAR) T-Cell in an immunotherapy setting, significantly destroying tumor cells in vitro [89, 90]. Furthermore, according to preclinical investigations, CAR T-cell therapy targeting FAP could be combined with cancer vaccines or immune checkpoint blockers such as anti-PD-1 and anti-CTLA4 and results in the blockade of some of the immunosuppressive factors such as DGKξ and TGF-β [90]. Another promising approach is using cancer vaccines that successfully target FAP. This approach could be implemented by three types of vaccines, including DNA, protein, and Dendritic Cell (DC) vaccines [91]. Vaccines elicit host immune responses by recruiting cytotoxic T cells against cancer cells [92]. Due to the higher stability of stromal fibroblast's genome than tumor cells, choosing these cells in immunotherapy could be more efficient than targeting tumor cell-specific antigens, especially in immunotherapies using DNA-based vaccines [89].

Antibody–drug conjugates are another tool in FAP immunotherapy targeting. For instance, OMTX705 is an antibody–drug conjugate developed by Fabre et al. An in vivo setting can elevate CD8^+^ T cell infiltration into the tumor, decrease tumor growth, and prolong the time of tumor recurrence [93]. OncoFAP in an ultra-high-affinity ligand of FAP was developed. It has a high affinity to bind FAP even in concentrations lower than nanomolar. According to the authors, it is well-tolerated and concurrently with a clinical-stage antibody-interleukin-2 fusion in curing tumor-bearing mice [94].

Prodrugs are another tool developed to deliver cytotoxic agents to their targets [95, 96]. They are a combination of peptides and enjoy low molecular weight, with the general structure of Z-Gly-Pro-Drug acting as substrates for the enzymatic activity of their specific target. Prodrugs cytotoxic agents are inactive until the target enzyme breaks it down and makes it less toxic than the original drug [95, 97]. Compared to the parent drugs, prodrugs are more soluble in water or lipid membranes and are better absorbed by target cells [98]. For example, Huang et al. developed a FAP-Targeting prodrug of Doxorubicin (FTPD) and conducted cytotoxicity analysis on 3T3 and HEK-293 cells. The developed FTPD was less toxic and safer than its original drug, doxorubicin, and it increased the therapeutic effects of doxorubicin [99]. Overall, FAP can be targeted through different approaches, such as CAR-T cell therapy, immunotherapy using vaccines, antibody–drug conjugates, prodrugs [97], and nano-drugs (e.g., BFO-PEG-FAP-inhibitor nanoconjugates) [100], FAP enzymatic activity inhibitors, and tumor suppressor microRNAs (e.g., MiR-30a) [97, 101].

### Expression of FAP and its correlation with disease prognosis in CRC

**Fig. 1:**
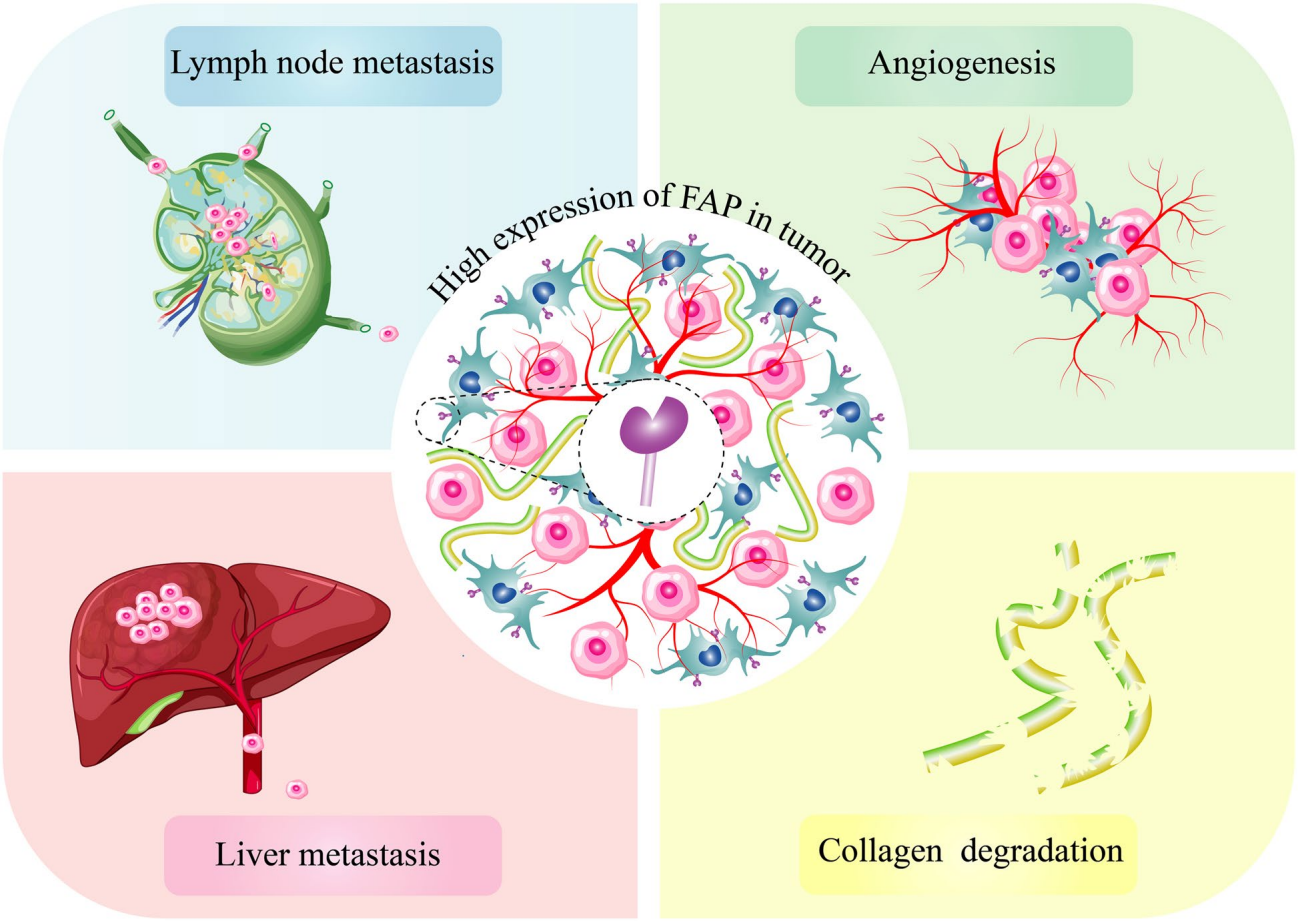
As proved in clinical and preclinical investigations, the overexpression of FAP in CRC will cause patient's poor prognosis, decrease in OS, increase in angiogenesis, elevates the rate of lymph node and liver metastasis, and induces collagen degradation in tumoral tissue

According to several studies, the overexpression of FAP in CRC is associated with TME remodeling, immunosuppressive effects [91], and more adverse clinical outcomes. These findings will be discussed in detail (Table 1**, **Fig. 1). Henry et al*.* conducted an IHC analysis to assess the expression of FAP in 138 sections of paraffin-embedded CRC tissues. They found that FAP was expressed in more than 93% of the samples. Also, a direct association was observed between FAP expression and poor survival in patients with metastatic CRC. They stated that patients with high stromal FAP expression face more aggressive disease and incidence of metastases or recurrence [41]. In another study, Chen et al*.* utilized IHC to evaluate the expression level of FAP in CRC samples collected before chemotherapy or radiotherapy from 60 patients. In vivo analyses on the immunological aspects of FAP overexpression showed that high FAP expression was associated with increased secretion of CCL2, recruitment of myeloid cells, and decreased activity of T cells that eventually caused immunosuppression in the CRC TME. Although this study has shown the effects of increasing FAP expression on the immune response and provided useful information in this field, the age of patients and their gender were not separated, and the role of age and gender in the survival of patients in two groups with FAP positive and FAP negative was not investigated [102]. Another study performed bioinformatic analysis using the publicly available Cancer Genomic Atlas (TCGA) and Gene Expression Profiling Interactive Analysis (GEPIA) to investigate the effect of FAP expression on CRC patients’ survival. They found that high FAP expression was associated with poor overall survival compared to patients with low FAP expression [103].

**Table 1:** The evaluation of FAP expression and its association with patients' clinicopathological factors

*n*	Method	Antibody against FAP	Impact of FAP overexpression on patient’s survival	Other Effects of FAP overexpression	Ref
92	IHC	Vitatex, Stony Brook, NY, USA	poor survival	Disease progression, advanced stages, angiogenesis, collagen degradation, and upregulation of genes involved in immune cell response The lymphatic invasion was more prevalent in FAP positive group in comparison to FAP negative group	[42]
127	SABC	United States Biological, Massachusetts, US	-	Increase in MVD, angiogenesis, A predictive marker for lymph node and liver metastasis	[64]
118	IHC	Novusbio, San Diego, CA	-	Venous invasion, perineural invasion, T status, lymph node metastasis, and N status were more prevalent in the intratumoral FAP positive group in comparison to FAP negative group	[104]
449	IHC	D8, Vitatex, Stony Brook, NY, USA	poor survival	Poor prognosis	[71]
52	Quantitative RT-PCR	-	poor survival	Increase distant recurrence and tumor re-growth after preoperative chemoradiation therapy	[43]
138	IHC	Monoclonal antibody D8	poor survival for patients with metastatic CRC	In xenotransplanted human CRC in mice, positively correlated with tumor progression, metastases, and recurrence	[41]
60	IHC	Abcam (CA, USA)	poor survival	Negatively associated with CD3^+^ cell number Correlated with an increase in CD11b^+^ cells number In CRC mouse model: induction of immune checkpoint blockade resistance	[102]
109	IHC	Dako, Carpinteria, CA, USA	-	Increase lymph node metastasis	[105]
	Immunofluorescence staining	AF3715 R&D, ab53066, ab28244 Abcam	-	FAP expression was higher in the invasive part in comparison to the tumor center	[106]
125	IHC	D8; Vitatex, Stony Brook, NY, USA	-	It is seen in the transformation of benign colorectal tissue into cancer In vitro: promotes the migration of CRC cells by producing FGF1	[39]
289	real-time PCR	-	poor survival	-	[107]

*IHC* Immunohistochemistry, *NY* New York, *CA* California, *USA* United States of America, *PCR* Polymerase Chain Reaction, *RT-PCR* Reverse Transcription-Polymerase Chain Reaction, *MVD* Micro-Vessel Density, *SABC* Street Avidin–Biotin Complex, *FGF1* Fibroblast Growth Factor 1

FAP expression in CRC stroma is positively associated with lymph node metastasis [38, 61–63]. Ma et al*.* evaluated FAP expression in 127 CRC, 51 colorectal polyps, and 28 normal tissues utilizing the immunohistochemical Street Avidin–Biotin Complex (SABC) method. They found that FAP expression was higher in CRC tissues compared to colorectal polyp samples. They concluded that FAP may play a role in CRC progression and tumorigenesis. The study showed that lymph node metastasis in the high FAP expression group was higher than in low FAP expression groups (60.43% vs. 33.4%, respectively). They also found a significant and positive association between FAP expression and Micro-Vessel Density (MVD) level meaning that FAP plays a role in CRC angiogenesis. They claimed that the extracellular matrix destructive power of FAP is why the FAP expression increases metastasis and invasion in CRC. This study is the only study that measured FAP expression in polyps. However, the relationship between FAP expression and tumor location has not been investigated. This study has not investigated the molecular mechanisms and pathways through which FAP affects clinicopathological characteristics [64].

Another study evaluated the mRNA and protein expression levels of FAP in 92 CRC tissue samples. A positive association was found between FAP expression, a higher disease stage, and poor survival. It was found that FAP expression promoted angiogenesis and collagen degradation. Moreover, higher expression of immune-cell process-related genes and higher frequency of macrophages and monocytes were noted in tissues having high FAP expression. The study suggested that increased angiogenesis and immunoregulation of TME may be induced by FAP expression in CRC stroma that eventually promotes tumor growth in this cancer.

It should be noted that this study was conducted with very small sample sizes, and the association between FAP expression and tumor location is not assessed [42]. FAP expression was evaluated in the tumor center and margin in 449 CRC tissue samples by IHC in another research. A positive association was noted between FAP expression in the tumor center and poor prognosis (multivariate hazard ratio, HR = 1.72, *p*-value = 0.025). In this study, Wikberg et al*.* investigated the difference in FAP expression in the center and periphery of the tumor and studied their relation to the prognosis of the patients. They reported increased FAP expression in the center of the tumor as a negative prognostic factor. They claimed that increased FAP expression in the tumor periphery was not associated with prognosis. Among the limitations of this study, it should be mentioned that, despite the high number of samples in the whole study, the number of samples in some subgroups was insufficient. Some of the patients in this study had received radiotherapy, and the statistics related to their prognosis were given along with others. Their data were not separated from the patients who did not receive radiotherapy. It cannot be separated from the effect of increasing FAP expression [71]. However, this association was not noted when FAP expression was high in the tumor margin. Nevertheless, unlike the tumor center, FAP expression in the tumor margin was associated with a higher tumor stage [71]. This finding was partially similar to the findings of Coto-Llerena et al*.*, which indicated that high FAP expression at the invasive margin positively correlates with the tumor stage; however, there is no such correlation regarding high FAP expression in the tumor center [42].

The mRNA expression level of FAP was also evaluated in 52 CRC patients who had undergone pre-operative chemoradiotherapy (CRT). The results did not reveal any correlation between stromal and serum FAP levels. The study concluded that it might be due to the localized activity of FAP as a cell-surface serine protease. Using Cox's univariate proportional hazards analysis, FAP high mRNA levels in residual cancer stroma after preoperative CRT showed a positive correlation with the incidence of tumor recurrence. In this study, the data before receiving chemo CRT is unavailable, so it is impossible to separate the effect of these treatments from the effect of increasing FAP expression on the prognosis of patients. Also, the sample size is insufficient in this investigation [43]. In another study, specific Enzyme-Linked Immunosorbent Assays (ELISAs) analysis was utilized to investigate the plasma levels of FAP in 47 CRC patients' plasma samples and 139 healthy volunteers. Cases with high plasma levels of FAP had worse survival than those with low plasma levels of FAP [108].

### FAP as a therapeutic target in CRC

**Fig. 2:**
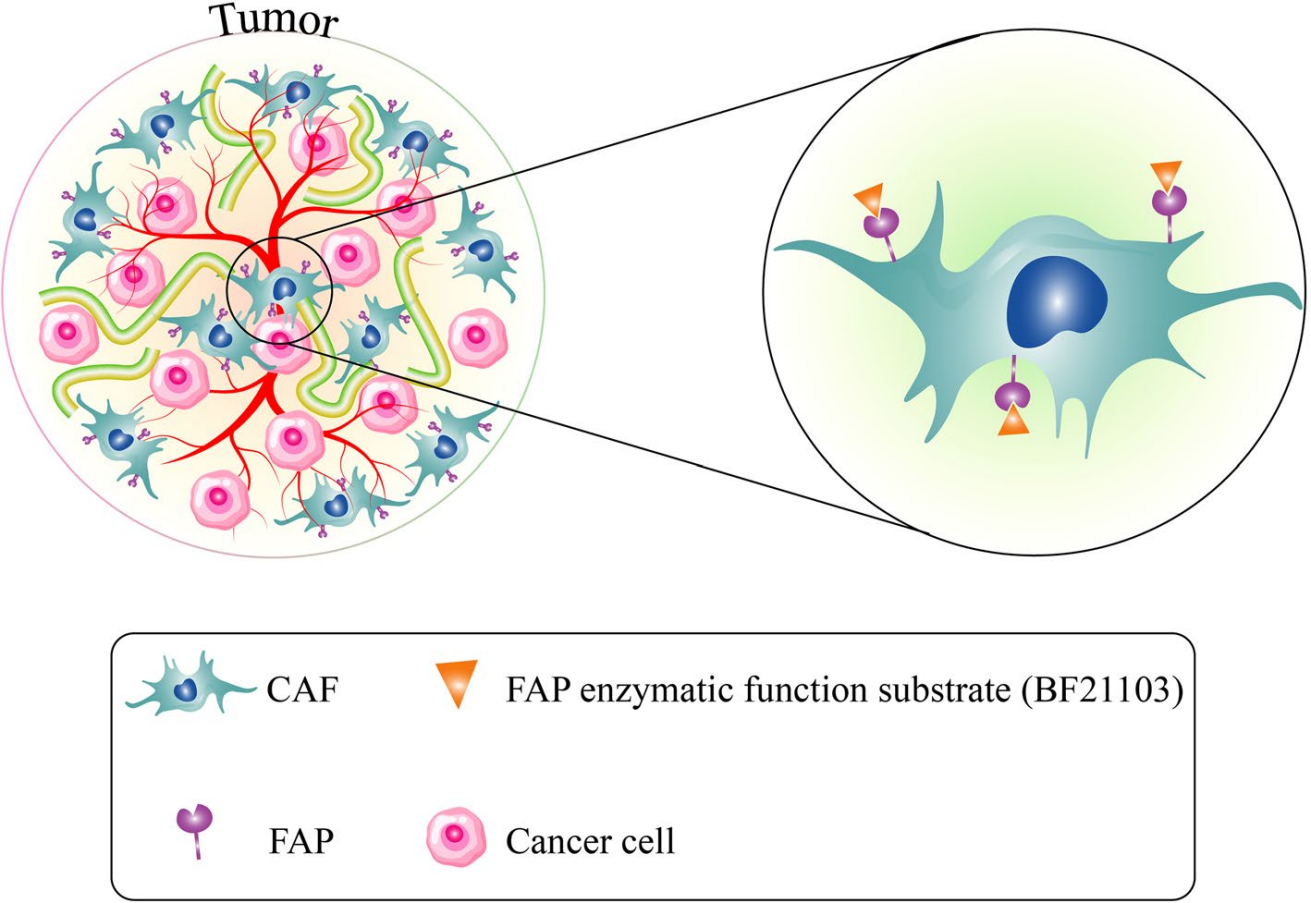
FAP enzymatic activity inhibition with BF211-03 as a substrate

Increased FAP expression in CRC stroma and its strong association with patients' prognosis has made this surface enzyme a promising target application for CRC diagnosis and treatment. FAP has been targeted in different approaches in CRC treatment, which will be discussed in detail (for further information, see also Tables 2 and 3) [109]. Narra et al*.* investigated the therapeutic effects of Val‑boroPro (Talabostat) in a phase II clinical trial in 28 patients suffering from metastatic CRC [110]. Talabostat is an orally active amino boronic dipeptide, inhibiting the FAP enzymatic activity [111, 112]. Despite the anti-tumor activity of Talabostat in different tumors, such as lymphoma, melanoma, mastocytoma, and fibrosarcoma, in vivo [113], the authors did not find any significant response or good clinical activity in their study [110]. Another study established a FAP-targeting prodrug, a substrate for FAP enzymatic activity. They synthesized a FAP-targeting traditional Chinese medicine-based prodrug named BF211-03, a prodrug of BF211 (a derivate of Bufalin). They investigated BF211-03 in human colon cancer HCT-116 xenografts and found that BF211-03 has tumor selectivity properties, and after cleaving by FAP, it successfully turned to BF211. In CRC xenograft models, BF211-03 showed anti-tumor activities and good stability in plasma and low heart and kidney toxicity (Fig. 2) [114].

**Table 2:** Inhibition of FAP in CRC treatment and its outcomes, preclinical phases

FAP inhibitor	FAP inhibitor type	Study type	Cell line/tumor model	Assay	Results	Refs
Oxytocin	peptide hormone	In vitro	Ls174t and SW480	Fluorescent IHC and invasion assay	Decrease in tumor cells migration	[115]
BF211-03	Dipeptide (Z-Gly-Pro)-conjugated BF211 prodrug	In vivo	HCT-116 xenograft model	Western blot, Cytotoxicity assay, and measuring body weight and tumor volume	Tumor selectivity and anti-tumor effects of prodrug were observed	[114]
*pcDNA3.1/V5-His-TOPO-Fap*	DNA vaccine	In vivo	BALB/c mice with CT26 cells, xenograft model	IHC and T cell infiltration	Increase host immune responses, CD8^+^ T cells activity, and survival while used in combination with chemotherapy	[16]
pFAP-vaccine	DNA vaccine	In vivo	CT26 mouse CRC xenograft model	IHC and cell staining	Primary tumor and pulmonary metastases suppression, promotion of T-cell-mediated host immune responses, increased survival	[89]
PT‑100 combined with oxaliplatin	dipeptidyl peptidase inhibitor and chemotherapeutic drug	In vivo	CT26 mouse CRC xenograft model	Western blot analysis, Flow cytometry, and RT‑qPCR	better the response to chemotherapy was observed, declined the activity of immune tumor‑promoting cells, inhibition of angiogenesis	[116]
M83	FAP/prolyl oligopeptidase inhibitor	In vivo	HCT116 mouse CRC model	IHC and Immunoblot Analysis	Decrease angiogenesis and tumor growth	[117]

*DNA* Deoxyribonucleic Acid, *RT‑qPCR* Reverse‑transcription quantitative polymerase chain reaction

**Table 3:** Clinical investigations regarding inhibition of FAP

FAP inhibitor	FAP inhibitor type	Study type	Sample size	Results	Refs
Sibrotuzumab	a humanized version of the murine anti-FAP mAb F19	Phase I open-label dose-escalation trial	20 Patients with CRC	No objective tumor response	[118]
unconjugated sibrotuzumab (BIBH 1)	a humanized version of the murine anti-FAP mAb F19	Phase I open-label, uncontrolled trial	25 Patients with metastatic CRC	Minimal clinical activity	[119]
Val‑boroPro (Talabostat)	inhibitor of dipeptidyl peptidases	phase II clinical trial	28 Patients with metastatic CRC	Minimal clinical activity	[110]

*mAb* Monoclonal Antibody

Another study evaluated an oral DNA vaccine targeting FAP in BALB/c mice transplanted with CT26 colon carcinoma cells after vaccination on days 3 and 10 after tumor injection. The results showed that this DNA vaccine stimulated immune response and promoted CD8^+^ T cell activity against tumor stromal cells expressing FAP. The expression of collagen type I was also significantly reduced in FAP-vaccinated mice. Moreover, when chemotherapy drugs were applied in FAP-vaccinated mice, the absorption of chemotherapeutic drugs became 70% higher, tumor growth was strongly suppressed, and the lifespan got much longer [16]. Similarly, it has shown that DNA vaccine against FAP in the CT26 mouse colon cancer model promoted anti-tumor immune responses by increasing the infiltration of CD8^+^ T cells and tumor lymphocytes into TME, decreasing the expression levels of collagen in TME, and prolonging survival. It must be noted that applying the FAP-targeting DNA vaccine did not affect wound healing or elicit autoimmune reactions. Furthermore, they used vaccination against FAP in a prophylactic setting in CT26 lung metastasis model mice and found that vaccination decreased the rate of pulmonary metastases incidence and increased survival [90].

FAP-based Whole-Cell Tumor Vaccine (WCTV) was developed in another study in which inactivated tumor cells expressed FAP protein. By injecting FAP-based WCTV, CRC xenograft models produced antibodies against FAP expressed on the surface of CAF cells. Further, FAP-based WCTV could have significant anti-tumor properties, slowing tumor growth and reducing the recurrence rate by eliciting host immune response in which antigen-specific cytotoxic T cells and CD4^+^ T lymphocytes participate. The study suggested that FAP-based WCTV could be an effective approach to target FAP by possessing significant therapeutic properties; however, the safety of this vaccine has not been appropriately investigated so far, and further studies are needed to explore its systematic toxicity profiles [120].

**Fig. 3:**
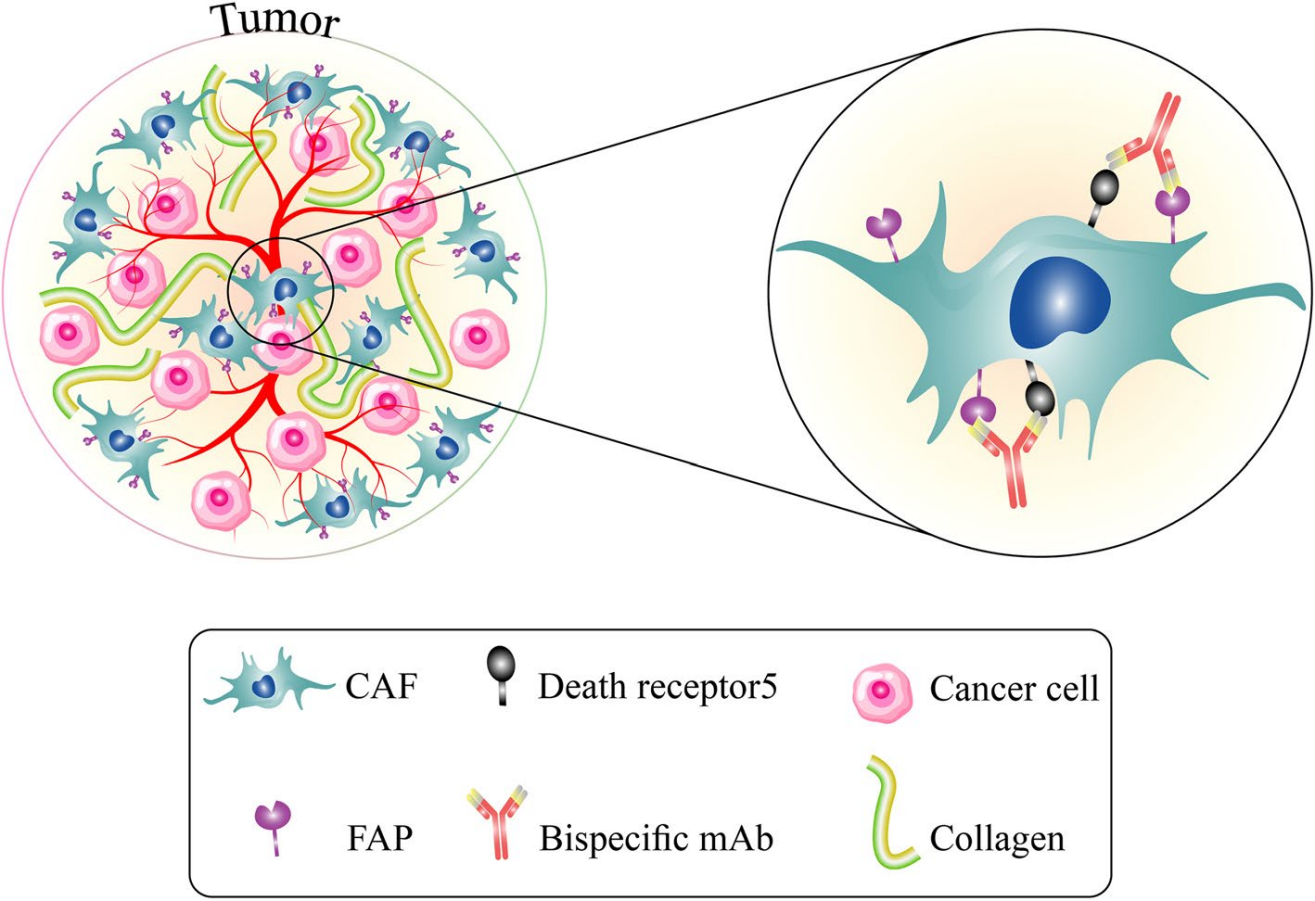
Dual targeting of FAP and death receptor 5 with a bispecific monoclonal antibody named RG7386. In vivo, it is demonstrated that RG7386 promotes apoptotic death in CRC cells

Monoclonal antibodies are another therapeutics being extensively used to treat different cancers. A phase I open-label dose-escalation trial was carried out to evaluate the safety, pharmacokinetics, and tumor uptake of Sibrotuzumab, a humanized version of the murine anti-FAP mAb F19. Sibrotuzumab was administered to 20 patients at a 5, 10, 25, or 50 mg/m^2^ dosage weekly and for 12 weeks. Six patients experienced adverse events due to the infusion of Sibrotuzumab. Gamma camera images were taken using [^131^I]-Sibrotuzumab. No absorption in normal organs was reported suggesting that Sibrotuzumab was a safe antibody; however, the authors stated that the vaccine failed to elicit a proper anti-tumor response [118]. In another study, Hofheinz et al*.* examined the therapeutic effects, safety, and pharmacokinetics of unconjugated Sibrotuzumab (BIBH 1) in 25 patients with metastatic CRC in an open-label, uncontrolled, multicentre trial. Unconjugated Sibrotuzumab was administered intravenously to patients at 100 mg for 12 weeks. They found that in 92% of the cases, the tumor continued to grow despite the drug infusions, and in the remaining, only a cessation of disease progression was observed. Five patients experienced adverse drug reactions such as rigors, chills, nausea, and flushing. The authors concluded that unconjugated Sibrotuzumab did not meet the minimum requirements for further clinical trials despite its patient safety and tolerability [119]. In another attempt, a bispecific antibody (BsAb) named RG7386 was developed to target FAP and death receptor 5 (DR5) concurrently. It was evaluated in vitro in the Colo205 cell line and in vivo investigations using patient-derived CRC FAP-positive stromal cells implanted in mice and CRC xenograft Co5896. RG7386 promoted the apoptotic process in these models. Brünker et al*.* also applied chemotherapeutic drugs, irinotecan or doxorubicin with RG7386 and found that this combination therapy effectively decreased tumor growth in CRC patient-derived xenograft models (Fig. 3) [121].

### FAP as a theranostic target in CRC

Molecular imaging targets specific molecules or structures and provides valuable information about biological processes at the molecular or cellular levels [122]. The history of targeted imaging for CRC goes back nearly 40 years ago when a radiolabeled monoclonal antibody targeting carcinoembryonic antigen (CEA) was used to localize CRC [123]. In this technique, gamma- or positron-emitter radionuclides are labeled with different ligands and administered to the patients. Then the kinetics of the radioligands is captured with the imaging equipment [124]. The same or at least similar ligands are labeled with beta- or alpha-emitters, providing therapeutic effects and constructing the theranostic concept, employing a ligand for diagnosis and therapy [125].

As discussed earlier, FAP is overexpressed in different malignancies, including CRC, aggravating the prognosis. FAP Inhibitors (FAPI) have been utilized to treat or control CRC; however, the preliminary results were unsatisfactory [110, 119]. Nevertheless, it still has the potential for theranostic application (Table 4). FAP became a target for nuclear medicine imaging in 1994 when Welt et al*.* used a FAP-targeting monoclonal antibody F19 labeled with Iodine-131 ([131I]I-mAbF19) for CRC imaging. 131I-mAbF19 was administered intravenously to 17 patients with hepatic metastases from colorectal carcinoma. No toxicity associated with intravenous 131I-mAbF19 administration was observed. The selective expression pattern of FAP allows imaging of colorectal carcinoma lesions as small as 1 cm in diameter on 131I-mAbF19 scans [126]. Besides gamma photons, Iodine-131 emits beta particles, which are also suitable for therapy. However, imaging with gamma-emitters has its inherent drawbacks. With the developments in nuclear medicine imaging instruments, Positron Emission Tomography (PET) became the main pillar of molecular imaging [122]. Moreover, radioligands with high tumor retention and rapid clearance from the rest of the body are also ideal for theranostic approaches. Some FAPI subtypes possess these fitting properties [127].

**Table 4:** Theranostic implications of FAP

Tracer	Radioisotope type	Therapeutic/ Diagnostic (imaging method)	Case number	Administered dose range	Findings	Refs
^68^ Ga-FAPI-04	Positron emitter	Diagnostic (PET)	38	122–312 MBq	Tracer absorption in tumor and image contrast was high	[128]
^68^ Ga-DOTA-FAPI-04	Positron emitter	Diagnostic (PET)	8	1.8–2.2 MBq	The diagnostic efficacy of the tracer was higher than [(18)F] FDG PET/CT	[129]
^68^ Ga-FAPI-04	Positron emitter	Diagnostic (PET)	13	1.8–2.2 MBq	The diagnostic efficacy of the tracer was higher than [(18)F] FDG PET/CT, and its absorption in the tumor was high	[130]
^68^ Ga-FAPI-2 and ^68^ Ga-FAPI-4	Positron emitter	Diagnostic (PET)	4	122–336 MBq	Regarding tumor-to-background contrast ratios, tracers had similar or even better efficacy than [(18)F] FDG	[131]
^68^ Ga-FAPI-04	Positron emitter	Diagnostic (PET)	15	111 to 298 MBq	Primary and metastatic tumors were detected by the tracer successfully. Tracer is a promising agent to determine the stage of lower GI system tumors	[132]
^177^Lu-FAP-2286	β-particle and Gamma emitter	Therapeutic and Diagnostic (SPECT)	1	5.8 ± 2.0 GBq	Tracer absorption and maintenance in the tumor were high and had a low toxicity	[133]
^68^ Ga-labeled FAPI	Positron emitter	Diagnostic (PET)	14 (including all GI system tumors)	52–325 MBq	Regarding tumor-to-background contrast ratios, tracers had similar or even better efficacy than [(18)F] FDG	[134]
^68^ Ga-FAPI-04	Positron emitter	Diagnostic (PET)	15	2–3 MBq/kg	The diagnostic efficacy of the tracer was higher than [(18)F] FDG PET/CT regarding the detection of liver metastases of GI system tumors	[135]

*DOTA* Dodecane tetraacetic acid, *MBq* Mega-becquerel, *PET* Positron emission tomography, *CT* Computed tomography, ^*68*^* Ga* Gallium-68, *[18F] FDG* [18F]fluorodeoxyglucose, *GBq*: Giga-becquerel, ^*177*^*Lu* Lutetium 177, *SPECT* Single-photon emission computed tomography

For oncology imaging, studies have shown promising results. The uptake of FAPI labeled with Gallium-68 ([68Ga]Ga-FAPI) showed intense uptake in different tumoral lesions and their metastases, including CRC [128, 129, 136]. Sollini et al*.*, in a meta-analysis, evaluated the diagnostic function of [68Ga]Ga-FAPI PET/Computed Tomography (CT) in different malignancies. Although this approach is not flawless, they reported a patient-based pooled sensitivity of 0.99 (95% Confidence Interval [CI], 0.97–1.00) and a pooled specificity of 0.87 (95% CI, 0.62–1.00) [137]. Their result indicated the significant diagnostic potential of [68Ga]Ga-FAPI PET/CT.

It must be noted that, currently, the evaluation of glucose metabolism in malignant cells, using [18F]fluorodeoxyglucose ([18F]FDG) is the most widely accepted radioligand imaging in oncology. Hence, the diagnostic performance of any new tracer is compared to [18F]FDG. In a recent systematic review, Treglia et al*.* reported that the target-to-background ratio of [68Ga]Ga-FAPI was higher than [18F]FDG, which may lead to better visualization per se and higher detection of smaller lesions, hypothetically. Additionally, they showed that the detection rate of [68Ga]Ga-FAPI PET/CT was equal to or higher than that of [18F]FDG PET/CT in different malignancies [138]. Being specific to CRC, the intensity of [68Ga]Ga-FAPI was also higher when compared to [18F]FDG [15], and [68Ga]Ga-FAPI PET/CT detected more lesions, especially in the case of disease recurrence [129, 130].

Another merit of [68Ga]Ga-FAPI is the low background uptake in the liver and brain [127, 128, 131, 136] that allows the detection of smaller hepatic metastasis. Moreover, non-invasive detection of tumoral cell depositions in the abdominal cavity (known as peritoneal carcinomatosis) is a challenge in GI malignancies. It has been shown that the intensity of uptake and sensitivity of [68Ga]Ga-FAPI PET/CT were higher than those of [18F]FDG PET/CT in detecting peritoneal carcinomatosis [139].

The ultimate aim of imaging is accurate management. The effect of [68Ga]Ga-FAPI PET/CT in lower GI tract malignancies was evaluated compared to the standard imaging. (68)Ga-FAPI PET/CT was performed on a cohort of 22 patients with LGT tumors, including 15 patients with metastatic disease, one with suspected local relapse, and six treatment-naïve patients. Uptake of (68)Ga-FAPI-04 and (68)Ga-FAPI-46 was quantified by SUV(max) and SUV(mean) after comparison with standard imaging. The highest uptake of FAPI tracer was observed in liver metastases and anal cancer, with an SUV(max) of 9.1 and 13.9, respectively. Because of low background activity in normal tissue, most lesions had a high tumor-to-background ratio of more than 3. Nothing was found in 47% of the patients (10/21), and the management significantly altered in 19% (4/21) [132]. The preliminary results from the clinical application of [68Ga]Ga-FAPI PET/CT were intriguing but needed future investigations to be confirmed. In another study, (68)Ga-FAPI uptake in primary tumors and metastases was comparable to (18)F-FDG in most cases. The SUV(max) was significantly lower for (68)Ga-FAPI than (18)F-FDG in background tissues such as the brain, oral mucosa, myocardium, blood pool, liver, pancreas, and colon. (68)Ga-FAPI TBRs were significantly higher than (18)F-FDG TBRs in some sites, including liver and bone metastases. Quantitative tumor uptake is comparable between (68)Ga-FAPI and (18)F-FDG, but lower background uptake in most normal organs results in equal or higher TBRs for (68)Ga-FAPI. Thus, (68)Ga-FAPI PET/CT may yield improved diagnostic information in various cancers and especially in tumor locations with high physiological (18)F-FDG uptake [134]. Şahin et al*.* compared the diagnostic performance of PET/CT imaging performed with (68)Ga-DOTA-FAPI and (18)FDG in detecting liver metastases in patients with gastrointestinal system cancer. They found that (68)Ga-DOTA-FAPI-PET/CT was superior over (18)FDG-PET/CT in the detection of liver metastases of GIS cancers, and it can be a complementary method, especially in negative cases with (18)FDG-PET/CT [135].

Non-specific uptake of [68Ga]Ga-FAPI is its main demerit. Except for tumors with significant expression of FAP, [68Ga]Ga-FAPI is accumulated in many other conditions, such as inflammatory processes [127, 129, 140–142], in which active fibrotic reaction is the main reason for false-positive findings.

**Fig. 4:**
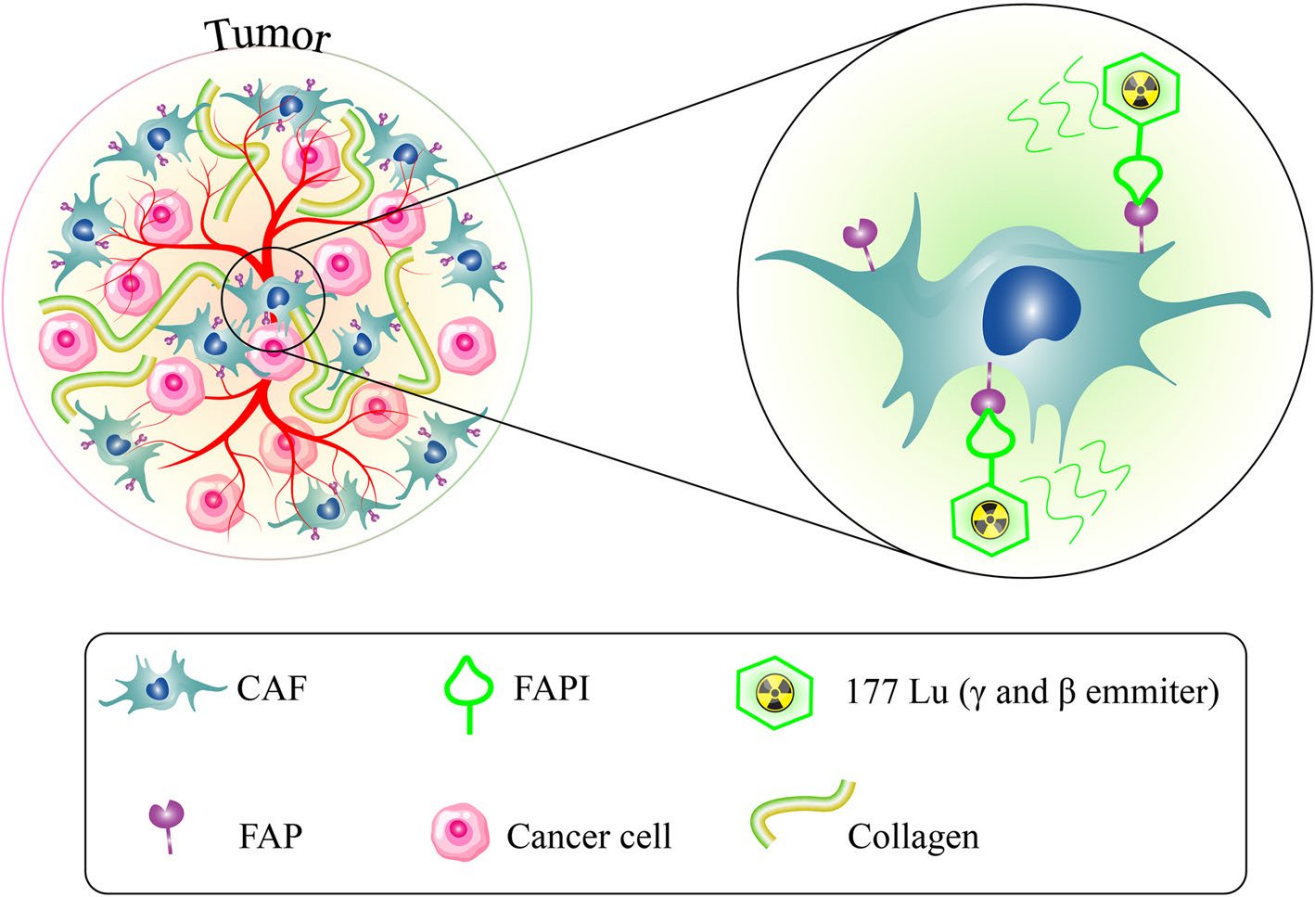
Targeting FAP with 177Lu-FAPI: a radiolabeled compound that binds to FAP and has antitumor activities via betta and gamma-ray emitting

There is an interest in radioligand therapy whenever a radiotracer successfully captures tumoral lesions. Radioligand therapy is usually considered when other standard treatments become ineffective. A limited number of studies about FAPI-based radioligand therapy are found in the literature review. Some therapeutic radionuclides, such as Copper-64 and Actinium-225, have been successfully labeled with FAPI, showing tumor-specific uptake [143]. In the clinical setting, Yttrium-90 and Lutetium-177 have been tagged with FAPI and administered in a few numbers of breast, ovarian, pancreatic, and thyroid cancers (Fig. 4) [127, 144–146]. Accordingly, a clinical investigation used (177)Lu-FAP-2286 in 11 patients with advanced adenocarcinomas of the pancreas, breast, rectum, and ovary after prior confirmation of uptake on (68)Ga-FAP-2286/-FAPI-04- PET/CT. They found that administration of (177)Lu-FAP-2286 (5.8 ± 2.0 GBq; range, 2.4–9.9 GBq) was well tolerated, with no adverse symptoms or clinically detectable pharmacologic effects. Significant uptake and long tumor retention of (177)Lu-FAP-2286 resulted in high absorbed tumor doses, e.g., 3.0 ± 2.7 Gy/GBq (range 0.5—10.6) in bone metastases [133]. Despite the absence of any significant adverse effects [127, 145, 146], the long-term outcome is yet to be determined.

The main challenge of FAPI-based radioligand therapy is its efflux from the malignant cells [143]. Using subtypes with longer retention in tumors and short-lived radionuclides is now conducted to overcome these disadvantages [127, 143].

In summary, FAPI-based molecular imaging provides valuable information on FAP expression in different malignancies, including CRC. Preliminary studies suggest that [68Ga]Ga-FAPI PET/CT enjoys high diagnostic performance than the current standard methods. Future studies should further clarify its role in staging and re-staging settings, prediction of prognosis, and effect on management. Moreover, FAPI-based radioligand therapy in advanced CRC patients is another intriguing aspect of targeted therapy, which is worth to be addressed in the following investigations.

## Conclusion

Concluding from the evidence and findings gained from the studies about the expression of FAP in CRC tumor samples and its association with prognosis. It can be said that high expression of FAP in CRC stroma increases angiogenesis, and collagen degradation in the tumor stroma elevates the rate of lymph node and liver metastasis and also increases disease recurrence. It also suppresses the immune system in the CRC microenvironment. FAP overexpression in CRC stroma is associated with poor patient overall survival and prognosis.

Despite many efforts to determine the extent of FAP expression and its association with the prognosis of CRC, there are still issues that need to be addressed in future studies. For instance, the association between FAP expression and patients' responses to radiotherapy and chemotherapy should be uncovered. Further studies are needed to investigate the association between tumoral site, age, smoking, and the patient's genetic history with FAP expression. Other studies could evaluate the role of FAP expression on the expression of other CAF markers, such as alpha-SMA, and elucidate the synergistic effect of these markers on prognosis in CRC. Moreover, measuring the expression of FAP in lymphatic and hepatic metastases samples help these types of metastases to be treated by targeting FAP in the future.

Considering FAP targeting in CRC, inhibiting FAP’s enzymatic activity with Talabostat does not bring an acceptable outcome in CRC treatment. However, prodrug BF211-03, a substrate for FAP, has shown good anti-tumor properties and low normal tissue toxicity. In immunotherapy with DNA vaccines, in vivo studies showed that vaccination against FAP triggers the host's immune response against the tumor, increases the penetration of CD8^+^ T cells into the TME, and reduces tumor growth. Altogether, the application of DNA vaccines against FAP in CRC is a promising approach needed to be more addressed in the future to clarify their clinical function and toxicity better. Regarding monoclonal antibodies, first, Sibrotuzumab was used to target FAP in CRC and did not bring promising clinical outcomes. Another study showed that a monoclonal antibody named RG7386, capable of simultaneously targeting FAP and DR5, had significant anti-tumor activities. This study confirmed that if properly designed, monoclonal antibodies could be promising nominees for targeting FAP in CRC; however, further studies are needed for more clarification.

FAP does not overexpress in all CRC patients; therefore, the decision to utilize appropriate therapies must be according to an initial assessment of FAP expression in each patient. In addition, FAP may express in non-tumor tissues such as wound healing sites, which makes it vital not to select a type of treatment that could affect the wound healing process.

Regarding FAP theranostic implications, FAPI-based molecular imaging provides helpful information about FAP expression in different malignancies, including CRC. previous studies suggest that [68Ga]Ga-FAPI PET/CT has higher diagnostic performance than the current standard methods. Future studies should clarify its role in staging and re-staging settings, prediction of prognosis, and effect on management. Moreover, FAPI-based radioligand therapy in advanced CRC patients is another intriguing aspect of targeted therapy, which is worth to be addressed in the following investigations.

## Data Availability

No experimental data used for writing this review article.
